# Automatic Localization of Stable Highest Dominant Frequency Area in AF Patients Based on Spatial Aggregation

**DOI:** 10.3390/bioengineering13050512

**Published:** 2026-04-28

**Authors:** Tao Huang, Yihang Jiang, Xiaomei Wu

**Affiliations:** 1 College of Biomedical Engineering, Fudan University, Shanghai 200433, China; 23210720076@m.fudan.edu.cn (T.H.); jungle_humst@foxmail.com (Y.J.); 2 Key Laboratory of Medical Imaging Computing and Computer Assisted Intervention (MICCAI) of Shanghai, Shanghai 200433, China; 3 Yiwu Research Institute of Fudan University, Yiwu 322000, China; 4 Shanghai Engineering Research Center of Assistive Devices, Shanghai 200433, China

**Keywords:** atrial fibrillation, atrial electrograms, catheter ablation, highest dominant frequency, spatiotemporal stability, spatial aggregation

## Abstract

Background: Atrial electrical activity in patients with persistent atrial fibrillation (PeAF) is extremely complex, and identifying optimal ablation targets during ablation procedures remains a significant challenge. This manuscript aims to identify spatiotemporally stable highest dominant frequency (HDF) in the left atrium to provide a reliable basis for Electrophysiologists to locate ablation targets beyond pulmonary veins. Methods: Filtering and spectral estimation were performed on the left atrial intracardiac electrogram (LA-EGM) of PeAF patients to recognize the dominant frequency (DF). Spatiotemporally stable DF features within the left atrium were extracted using spatial aggregation and others to construct a 3D DF distribution model. HDF areas were automatically identified based on personalized thresholds derived from the patient’s DF distribution. Results: Data analysis of 43 PeAF patients demonstrated that spatial aggregation with 2 mm voxel size accurately constructs spatiotemporally stable DF distribution models. The proposed DF model enables the automatic identification of stable HDF areas in PeAF patients. In retrospective clinical cases, 72.1% of patients underwent ablation at these identified sites with effective therapeutic outcomes. Conclusion: Recurring HDF areas during PeAF serve as potential ablation targets. The results of this study provide a reliable basis for determining personalized ablation targets for PeAF patients.

## 1. Introduction

Atrial fibrillation (AF) is one of the most common cardiac arrhythmias in clinical practice, characterized by rapid and uncoordinated atrial electrical activity. Globally, 2–4% of people suffer from AF, and the number of affected individuals is increasing yearly [1]. The incidence of AF increases with age [2], and with the global population aging, it is estimated that the total number of AF patients will double by 2050 [3]. AF can lead to thrombus formation, increasing the risk of stroke, systemic thromboembolism, and vascular dementia, and it can induce heart failure and other heart diseases [4]. Ref. [5] proposed a novel therapeutic approach for AF by designing a cardiac patch to restore electrical conduction between myocardial tissues obstructed by scar tissue. In mainstream clinical practice, catheter ablation based on circumferential pulmonary vein isolation (PVI) is the primary treatment for AF, yielding favorable outcomes in paroxysmal AF. However, due to the complex arrhythmic mechanisms of persistent atrial fibrillation (PeAF), identifying extra-pulmonary vein ablation targets in patients with PeAF remains a significant challenge [6]. Several studies have explored the mechanisms underlying arrhythmias. Ref. [7] identified that glucagon-like peptide-1 receptor (GLP-1R locus) agonists can reduce the risk of certain arrhythmias. Ref. [8] discovered that moderate mitophagy exerts a protective effect on myocardial tissue. Ref. [9] investigated the poorly understood mechanisms of heart failure and identified 14 significant genes associated with the condition.

Previous studies have shown that PeAF may induce tissue reconstruction and electrical reconstruction in the atrial tissue [10,11]. These reconstruction regions may harbor underlying localized abnormal electrical potential activity, leading to local fibrillatory conduction and the formation of re-entry circuits, which are crucial for triggering and maintaining AF. In the treatment of AF, ref. [12] utilized Complex Fractionated Atrial Electrograms (CFAEs) as targets for catheter ablation, achieving certain clinical efficacy. Ref. [13] calculated spatiotemporal dispersion features from EGM and performed ablation on regions exhibiting such dispersion, demonstrating clinical effectiveness. Furthermore, ref. [14] designed the first large-scale international positive randomized controlled trial (RCT) in PeAF, where ablation of spatiotemporal dispersion areas in patient EGM yielded significant clinical outcomes.

The dominant frequency (DF), as a critical feature extracted from EGMs, plays a vital role in the treatment of AF. DF is the most prominent frequency component in the local electrical potential signal of the atrial tissue. By performing frequency analysis and calculating the DF from the acquired 3D atrial electrical mapping signals, the core frequency characteristics of the electrical activity in the relevant atrial regions can be precisely captured. Unlike normal sinus rhythm, the DF distribution within the atrium during AF is generally not uniform but has a certain gradient and significant inter-regional differences. The HDF area is the most electrically active and strongest driving region within the atrium. Some studies [15,16] suggest that the HDF area plays a vital role in the onset and maintenance of PeAF and is a potential target for AF ablation.

Studies by [17,18] showed that after PVI, ablating the HDF areas in patients with PeAF increased the patient’s long-term cure rate from less than 60% to over 80%. However, other studies [19,20] found no significant difference compared to the control group after ablating the HDF areas in patients with PeAF. This wide disparity in results is primarily due to the highly heterogeneous nature of atrial electrical activity in PeAF patients. Within the abnormal electrical activity regions formed by atrial tissue remodeling, some HDFs are not active abnormal electrical activity sources but are transient, high-frequency “passive excitations” that randomly change with overall electrical activity disorder. Only spatiotemporally stable HDF areas are highly coupled with atrial tissue remodeling and electrical remodeling and are closely related to the onset and maintenance of AF. The research of [19,20,21,22] also confirmed that spatiotemporally stable HDF areas overlap to some extent with the site of rotors.

The current mainstream atrial electrical mapping methods involve moving a contact electrode within the atrium to sequentially acquire Intracardiac Electrogram (EGM) signals and electrode position coordinates, known as sequential mapping [21,23]. This mapping method can yield good results under relatively stable atrial electrical activity. However, under AF conditions, the DF obtained by frequency domain analysis of EGM signals collected by sequential mapping using a fixed window width is instantaneous and cannot truly reflect the core frequency characteristics of the local electrical activity. Ablation of such HDF areas is often ineffective [24,25].

To address the aforementioned issues, this study proposes a method for automatically localizing spatiotemporally stable HDF areas in PeAF patients based on sequentially mapped Left Atrial Intracardiac Electrogram (LA-EGM) signals. This algorithm generates a three-dimensional (3D) DF distribution model based on left atrial mapping data acquired during ablation, enabling the localization of spatiotemporally stable HDF areas. During PeAF ablation procedures, this algorithm provides reliable auxiliary basis information to assist Electrophysiologists in formulating ablation strategies. The main contributions of this paper are as follows:

- A spatial aggregation method for extracting spatiotemporally stable HDF is proposed. Through time-domain sparse sampling and local neighborhood spatial filtering, the computational load was effectively reduced and the robustness to abnormal signals was enhanced.
- The significance of the DF is effectively enhanced by calculating the Organization Index (OI) threshold, and an experimental paradigm for reasonably setting the OI threshold based on specific data distribution is provided.

The structure of this paper is as follows: Section 2 introduces the data used in the experiment and the details of the proposed method. Section 3 and Section 4 present the results of the generated spatiotemporally stable DF distribution model and discuss the parameters and effectiveness of each method, as well as the method’s impact on ablation planning. Finally, the limitations of this study are discussed, and the conclusions of this research are presented.

## 2. Materials and Methods

**Figure 1:**
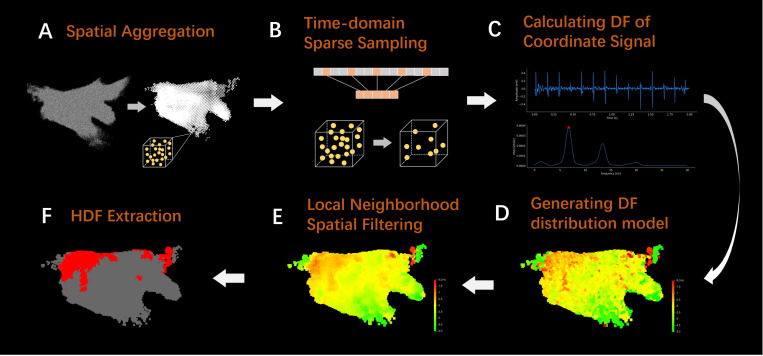
Flowchart of atrial electrogram data processing. (**A**) Schematic diagram of spatial aggregation. (**B**) Schematic diagram of time-domain sparse sampling. (**C**) The amplitude and spectrum of signal when calculating DF. (**D**) Generating DF distribution model. (**E**) The result of local neighborhood spatial filtering. (**F**) The result of HDF extraction.

The research framework of this manuscript is shown in Figure 1. To obtain spatiotemporal stability DF information at local positions in the left atrium, the LA-EGM data is first subjected to spatial aggregation to obtain voxel units (Figure 1A), effectively solving the problem of unstable DF information obtained by directly processing sequential mapping data. Subsequently, time-domain sparse sampling is performed on the mapping coordinates within the voxel unit (Figure 1B) to significantly reduce the computational cost of the model. When calculating the DF for each coordinate point within the voxel unit (Figure 1C), the OI is used to ensure the significance of the DF. Then, a three-dimensional (3D) DF distribution model is constructed based on the aggregated voxel unit position and corresponding DF values (Figure 1D), and local neighborhood spatial filtering is applied (Figure 1E) to eliminate the interference of abnormal DF values on the model. Finally, the HDF area is extracted based on the overall DF distribution of the model (Figure 1F), providing a reliable reference basis for clinical determination of personalized ablation targets.

### 2.1. Dataset and Preprocessing

The proprietary dataset for this study comes from clinical LA-EGM signal data collected during catheter ablation procedures performed on PeAF patients across four hospitals. During the procedure, the Electrophysiologist placed a 10-electrode catheter (CS) via the femoral vein access into the patient’s coronary sinus to acquire global atrial activation signals, serving as the standard for determining if the patient was in AF. Subsequently, a star-shaped OCT catheter equipped with 22 electrodes (Apt Medical Inc., Shenzhen, China) was deployed in the left atrium via venous access to perform sequential mapping. Using an electro-anatomical mapping system, the Electrophysiologist performed approximately 30 min of left atrial mapping on 43 PeAF patients (28 males, average age 66.37 ± 9.44 years, 15 females, average age 68.64 ± 7.27 years) before ablation. LA-EGM (sampling frequency is 1000 Hz) signals were collected from the left atrial endocardium, and the electrode coordinate sampling frequency was 40 Hz. The model of the CS catheter was 901667, with an inter-electrode spacing of 2-8-2-8-2-8-2-8-2 mm, and the OCT catheter had an inter-electrode spacing of 2-5-2 mm. The ethical approval numbers for the clinical trial are NO.:QX-2022-001-01 and Equipment clinical review [2022] No. (04).

This manuscript retrospectively analyzes the LA-EGM signals collected by the OCT catheter during the aforementioned clinical procedures. The bipolar voltage between adjacent electrodes was calculated, and 15 effective long-sequence bipolar LA-EGM signals were extracted. A peak detection method [26,27] was used to recognize the activation peaks of the bipolar LA-EGM signals collected by the CS and calculate the atrial activation cycle length, thus recognizing periods with abnormal activation cycles to locate the start and end times of AF. Based on the AF periods located by the CS signal, the LA-EGM signals collected by the OCT catheter during AF were extracted for subsequent computational analysis in this study. Since clinical mapping equipment pre-filters the atrial EGM signals, this study primarily focuses on identifying and removing abnormal signals: ① removal of random noise with amplitudes below 0.2 mV; ② removal of artifacts with amplitudes exceeding 16 mV; ③ removal of signal segments with excessive power line interference through energy distribution analysis of the signal spectrum via FFT.

### 2.2. Spatial Aggregation

Adopting a fixed window width for time-frequency analysis of LA-EGM signals collected by sequential mapping only provides DF information for a local position at a single moment. Such DF is not necessarily stable and cannot be used to determine potential ablation targets. Furthermore, sequential mapping typically results in an over-sampling of signals in complex left atrial regions (such as the pulmonary vein ostia) and insufficient sampling in simple regions (such as the left atrial anterior wall), leading to an uneven spatial distribution of signal density in the constructed 3D DF model, which affects model performance. To address this, this manuscript proposes a voxel-level spatial aggregation method, as shown in Figure 1A, which aggregates the information from all mapped electrode coordinate points within a certain range to calculate the DF and ultimately construct a complete left atrial DF spatial distribution model.

During the spatial aggregation process, it is first necessary to determine the spatial range of a 3D cube that completely encloses all the anatomical structures of the left atrium, as well as the coordinate points of the pulmonary vein regions closely related to left atrial electrical activity. After repeated experimental calculations and boundary verifications, the spatial range of this cube was finally set to 100 mm × 100 mm × 100 mm. Subsequently, the cube is uniformly divided into several small cubic blocks with a side length of 2 mm. Each small block is defined as a voxel unit, and the centroid of the voxel is used as the 3D coordinate position of the unit, allowing for LA-EGM signal integration with the voxel unit as the basic unit.

When integrating the LA-EGM signals for each voxel unit, the LA-EGM signal segment corresponding to the sampling time of each coordinate point is extracted, the DF of that signal segment is calculated, and this is used as the DF value for that coordinate point (the specific method for calculating DF will be detailed in Section 2.4).

In the sequential mapping process, due to the uncertainty of the electrode catheter’s sliding path, the sampling times of the coordinate points within the voxel unit exhibit a large time span (approximately 28 min on average). This characteristic implies the dynamic change feature of the myocardial electrical activity at that location over a long period. Fully utilizing this information allows for reflection of the DF’s stability over a long time span at that location. When calculating the voxel unit DF, the DF values corresponding to all coordinate points within the voxel are first sorted in ascending order to obtain a DF sequence, where n is the number of coordinate points within the voxel. Then, the Trimmed Mean of the DF sequence is calculated using Equations (1) and (2) as the DF value for that voxel unit.

(1) k=αn

(2) $$x_{trim}=\frac{1}{n-2k}\sum_{i=k+1}^{n-k}x_{i}\;$$

where α (0≤α<0.5) is the trimming proportion parameter, n is the length of the array, k is the number of values trimmed, and $x_{trim}$ is the Trimmed Mean DF of the voxel unit. This method allows for the removal of overly large or small DF values caused by various uncertain factors, such as catheter contact, yielding a DF value that can more accurately characterize the electrical activity features of the region.

### 2.3. Time-Domain Sparse Sampling

The spatial aggregation method described in Section 2.2 may lead to an excessive number of coordinate points in corresponding voxel units due to overly dense sampling in some regions. If the DF is calculated for every single coordinate point, the computational efficiency will be significantly reduced. This manuscript proposes a sparse sampling method in the time domain to sparsely sample the coordinate points within the voxel unit. By filtering based on the coordinate points’ distribution characteristics in the time domain, the DF is calculated only for the selected subset of coordinate points, thereby effectively improving computational efficiency while preserving the temporal span of the signal.

**Figure 2:**
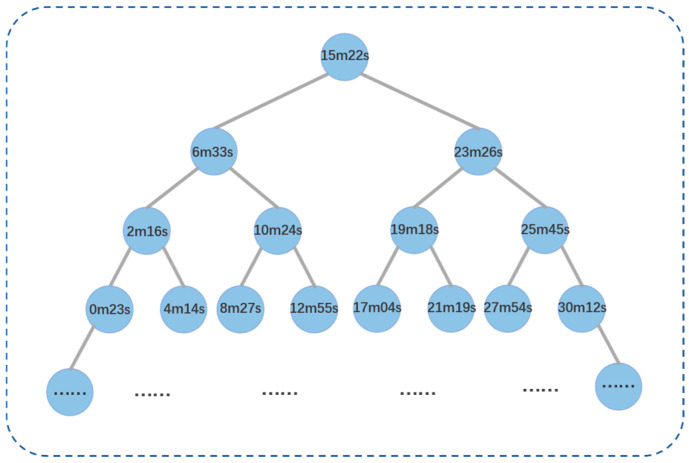
Binary search tree for time-domain sparse sampling.

The specific steps for sparse sampling in the time domain are as follows: ① Sort all coordinate points contained in the voxel unit in ascending order according to their mapping time. ② Construct a binary search tree, as shown in Figure 2, with the coordinate point at the middle position of the coordinate point sequence as the root node. The value of each node in the tree is the time value of the corresponding coordinate point. ③ Based on the characteristic that the values of all nodes on the left subtree are less than the root node and the values of all nodes on the right subtree are greater than the root node, perform a Breadth-First Search traversal on the constructed binary search tree. Nodes traversed during the process are sequentially added to a queue until the queue length reaches 31 (an empirical value). By constructing a binary search tree and performing a Breadth-First Search traversal, an approximately equal-interval fast sampling of the coordinate point sequence in the time domain can be achieved. The setting of the queue length should balance the time-domain resolution after sampling with computational efficiency.

### 2.4. Calculating DF of Coordinate Signal

After completing spatial aggregation and time-domain sparse sampling, the corresponding DF value for each coordinate point contained within the voxel unit needs to be calculated. This study uses the signal segment within a 2-s time window centered around the mapping time of the coordinate point as the corresponding LA-EGM signal for that point. First, signal segments with amplitudes less than 0.2 mV and greater than 16 mV are filtered out as noise. The signal segment is then subjected to Fourier transform and spectral analysis to discard power-line interference components. Next, the filtered signal segment is filtered based on the Botteron method [3]: ① 40–250 bandpass filtering; ② rectification; ③ 20 Hz low-pass filtering. Subsequently, power spectral analysis is performed on the filtered signal segment using Welch [28], utilizing a Hamming window to reduce leakage, a 50% overlap, and a frequency step set to 0.05 Hz. The Welch method incorporates segmentation, windowing, overlapping, and averaging to mitigate the influence of localized amplitude anomalies in EGM on the global power spectrum, thereby enhancing the stability of the results. Within the power spectrum, the DF value is defined as the spectral peak within the physiological signal energy range of 4–10 Hz in the calculated power spectrum [6].

To better address the influence of wavefront fusion and signal quality on DF calculation, and to distinguish between the DF and its harmonics, this manuscript assesses the significance of the recognized DF peak by calculating the OI value [29,30]. The OI is the ratio of the area of the DF and its harmonics to the total area of the power spectrum within the 0–20 Hz frequency range. The calculation for the value of the signal’s power spectrum X(w) is shown in Equation (3):

(3) $$OI=\frac{\sum_{w=w_{df}-k}^{w_{df}+k}X\left(w\right)+\sum_{j=1}^{N}\sum_{w=h_{j}-k}^{h_{j}+k}X\left(w\right)}{\sum_{w=w_{l}}^{w_{h}}X\left(w\right)}\;$$

where $w_{df}$ is the recognized DF value, $h_{j}$ is the frequency value corresponding to the harmonic peak of the DF, *k* is the DF width threshold (set to 0.375 Hz) [24], and *N* is the total number of harmonic peaks within the specific frequency band (in this manuscript, $w_{l}$ = 0 Hz, $w_{h}$ = 20 Hz).

After calculating the power spectrum and the DF value corresponding to the signal segment, the OI value of the DF peak is calculated. An OI threshold of 0.2 is set. DF peaks with an OI value less than 0.2 are considered to have insufficient significance and thus cannot represent the activation frequency of the local atrial tissue, so they are filtered out.

### 2.5. Local Neighborhood Spatial Filtering

After completing the signal processing described in Section 2.2, Section 2.3 and Section 2.4, a 3D DF distribution model can be constructed based on the voxel coordinate positions and the Trimmed Mean DF of the voxels. However, potential factors during cardiac electrophysiological mapping, such as poor electrode-myocardium contact and environmental noise interference, can cause errors in the DF calculation for some coordinate points, affecting the accuracy of the voxel unit point cloud DF values. To address this, this manuscript proposes a Local Neighborhood Spatial aggregation method. The core idea is to find the 10 nearest neighboring voxel units in 3D space for each voxel unit in the 3D DF model, calculate the average DF value of that voxel unit and its neighbors, and use this average value to update the DF value of that point. This method effectively suppresses DF outliers caused by random interference, allowing the DF’s spatial distribution to present a more natural and coherent form that aligns better with the physiological characteristics of myocardial electrical activity.

To achieve efficient searching for neighboring voxel units, this study uses a kd-tree [31] as the data structure for storing the 3D coordinate point cloud. When constructing the kd-tree, the median point of all voxel units’ 3D coordinates is used as the root node. At each level, one coordinate dimension is bisected: voxel units smaller than the boundary threshold are assigned to the left subtree, and those larger are assigned to the right subtree. The coordinate dimension used for splitting at each level is rotated, following the sequence of x-axis, y-axis, and z-axis. Upon final partition, each subtree contains only one voxel unit, forming a complete spatial indexing structure.

**Figure 3:**
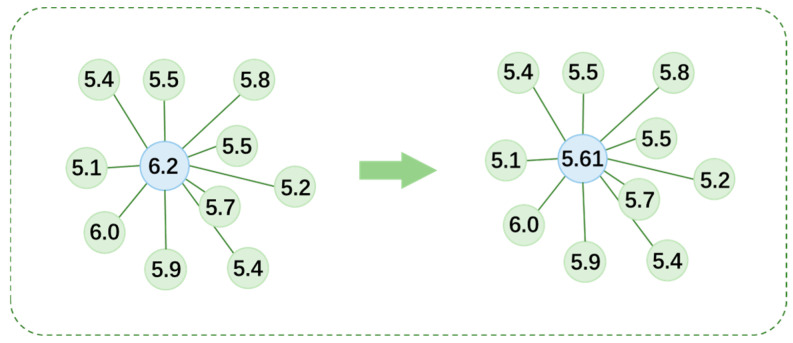
Schematic diagram of the structure of local neighborhood space filtering.

Once the kd-tree is constructed, it can efficiently find the 10 nearest neighboring voxel units for each voxel unit. This selective traversal strategy significantly improves search efficiency compared to a global search. As shown in Figure 3, after successfully obtaining the 10 nearest neighboring voxel units for the blue voxel unit, its own DF value and the DF values of these 10 neighboring voxel units are arithmetically averaged. The resulting value is the new DF value for that voxel unit after the Local Neighborhood Spatial aggregation processing.

### 2.6. HDF Extraction

After calculating the DF value for each voxel unit according to the aforementioned procedure, a 3D DF distribution model can be constructed by combining the voxel unit’s spatial coordinates with its corresponding DF value. Since the intrinsic dominant frequency of PeAF patients exhibits significant inter-individual variation, using a fixed frequency threshold to delineate the HDF area may lead to biased results. Therefore, to enhance the individual adaptability and accuracy of the analysis, this study sets a personalized threshold based on the patient’s own DF distribution; the HDF area is defined as the region constituted by coordinate points whose DF value is higher than the mean DF of all voxel units plus one standard deviation [32]. On this basis, all voxels undergo binary labeling: DF values above this threshold are labeled as 1, and those below the threshold are labeled as 0, thereby precisely distinguishing the HDF area from the non-HDF area and providing a spatial reference that better aligns with individual characteristics for subsequent clinical analysis.

## 3. Results

This manuscript retrospectively analyzed LA-EGM data collected during radiofrequency ablation procedures from 43 PeAF patients across four hospitals. The corresponding 3D DF distribution models were generated, and the HDF area within them was extracted and analyzed.

### 3.1. Results of Signal Processing and HDF Extraction

**Figure 4:**
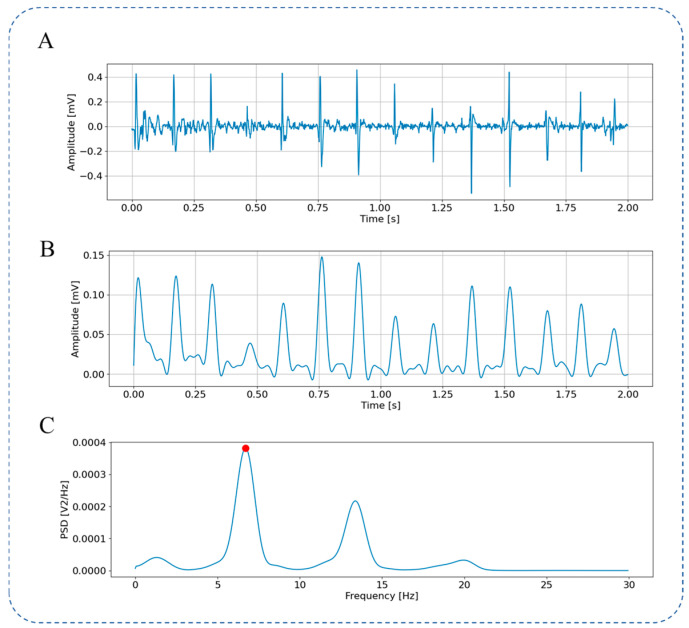
LA-EGM Signal Preprocessing. (**A**) Original LA-EGM signal; (**B**) LA-EGM signal filtered by Botteron; (**C**) spectrum ranging from 0 to 30 Hz obtained through Welch.

Figure 4 shows the raw LA-EGM signal (Figure 4A) and the pre-processing steps. It can be observed that Botteron filtering converts the atrial activation signal into a pulse activation sequence, as shown in Figure 4B. This filtering removes the fine-grained features of individual atrial activations and highlights the temporal characteristics of the activation, facilitating the analysis of the atrial activation frequency. The signal power spectrum calculated using the Welch from 0 Hz to 30 Hz is shown in Figure 4C. A peak is visible around 6.7 Hz (marked by the red point). The frequency corresponding to this peak is the DF of this coordinate point.

**Figure 5:**
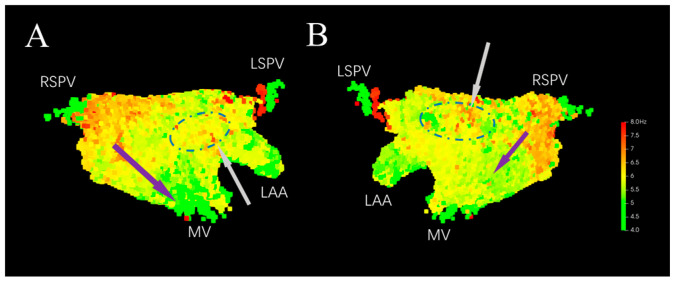
Original 3D DF distribution model. (**A**) Anterior–Posterior. (**B**) Posterior–Anterior. LSPV: Left Superior Pulmonary Vein. RSPV: Right Superior Pulmonary Vein. LAA: Left Atrial Appendage. MV: Mitral Valve.

As shown in Figure 5, the 3D DF distribution model is constructed based on the 3D coordinate positions of the voxel units and their DF values. From Figure 5A, the DF can be seen exhibiting a gradient distribution along the direction indicated by the purple arrow in the 3D model, with a higher value near the right pulmonary vein and gradually decreasing near the mitral valve. From Figure 5B, the DF value is also higher near the right pulmonary vein, with a smaller gradient towards the inferior wall. DF values are higher in the middle of posterior wall and at the junction of the left atrial appendage and the anterior wall, showing a focal distribution (indicated by the gray arrows and blue dashed circles in Figure 5).

**Figure 6:**
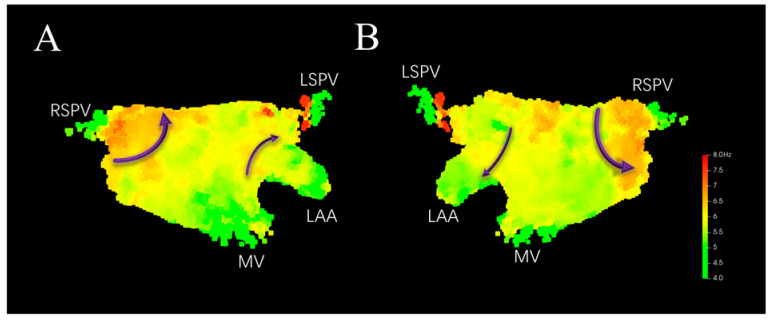
3D DF distribution model after local neighborhood spatial filtering. (**A**) Anterior–Posterior. (**B**) Posterior–Anterior. Purple arrows indicate potential re-entry paths.

Applying Local Neighborhood Spatial aggregation filtering to the model shown in Figure 5 yields Figure 6. It can be seen that locally abnormal DF values at corresponding positions are filtered out (indicated by the dashed blue circles in Figure 5). The distribution of DF on the left atrial surface is smoother, with the DF showing a gradual descent from high to low, which helps in more accurately capturing the HDF sites. The DF values are higher around the right pulmonary vein, where re-entry circuits around the vein ostium may exist. Higher DF values near the anterior wall close to the left atrial appendage and in the center of the posterior wall may indicate the presence of micro-reentry (indicated by the purple arrows in Figure 6).

**Figure 7:**
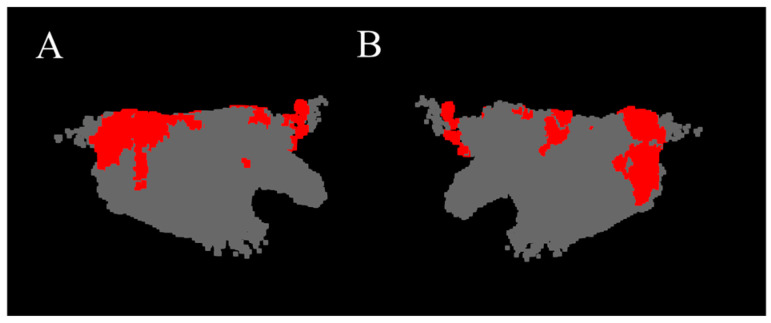
HDF area of the 3D DF model (red region). (**A**) Anterior–Posterior. (**B**) Posterior–Anterior.

The red regions in Figure 7 show the HDF area constituted by coordinate point clouds whose DF value is higher than the mean plus one standard deviation. It can be seen that in this case, the HDF is mainly distributed at the junction of the left pulmonary vein and the interatrial septum, in the center of the posterior wall, and sparingly at the junction of the anterior wall and the left atrial appendage.

### 3.2. Result Validation

All patients included in this study underwent radiofrequency ablation therapy. During the procedure, PVI was initially performed as the foundational ablation step. Subsequently, AF termination was assessed via induction; if AF persisted, further linear ablation was implemented based on electroanatomical mapping results. Following this, induction was repeated to observe AF termination; if AF continued, potential drivers such as rotors or CFAEs were localized through mapping, and focal ablation was performed in the corresponding regions. Additionally, substrate modification ablation was performed in some patients based on atrial substrate characteristics.

To validate the relationship between the HDF area extracted by this manuscript and the ablation targets, the HDF area in the generated 3D DF models was compared with the ablation sites during the ablation surgery of PeAF patients. In the data from 43 patients, HDF area outside the pulmonary vein ostia existed in the 3D DF models of 38 patients. Among these, the effective ablation targets outside the circumferential pulmonary vein isolation in 31 patients corresponded to the HDF area extracted by this manuscript. After the Electrophysiologist ablated these HDF areas, AF was terminated.

**Figure 8:**
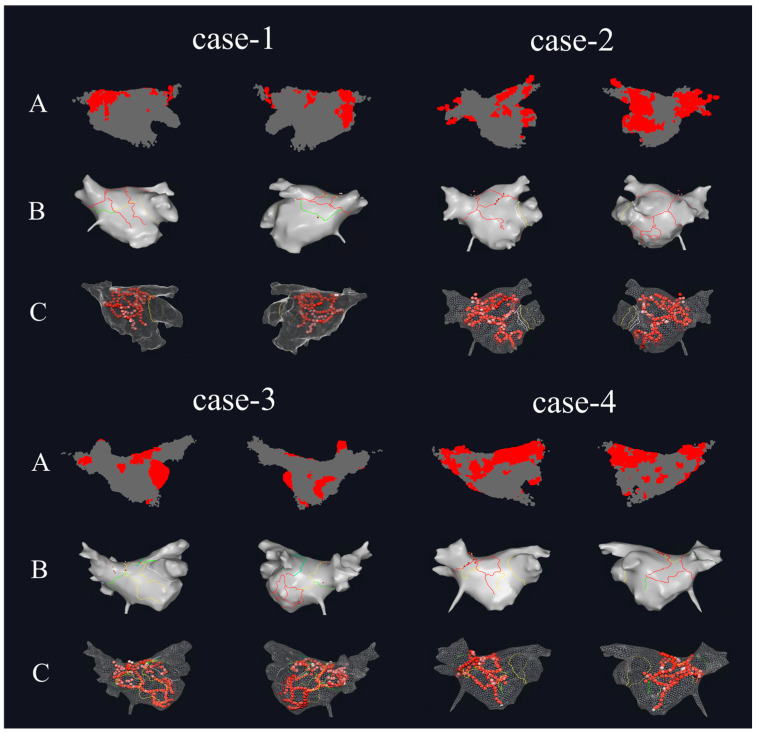
The comparison between the HDF regions calculated by this paper and the actual surgical ablation targets. (**A**) The HDF area of the 3D DF model of PeAF patients (left side is Anterior–Posterior, right side is Posterior–Anterior). (**B**) The intraoperative ablation planning for PeAF patients (left side is Anterior–Posterior, right side is Posterior–Anterior). (**C**) The distribution of ablation targets for PeAF patients (left side is Anterior–Posterior, right side is Posterior–Anterior).

Figure 8 shows the HDF area in the 3D DF models and the actual ablation situations during the surgical procedure for four of the PeAF patients. Figure 8A is the 3D DF model generated based on the patient’s atrial mapping data using the method proposed in this study. It can be seen that in the 3D DF model of case-1, the HDF area is mainly distributed on the Septum wall, posterior wall, and inferior wall near the left pulmonary vein, with a small distribution on the anterior wall near the left atrial appendage region. The HDF area in case-2 is mainly distributed at the two inferior pulmonary vein ostia and the posterior side of the left atrial appendage near the mitral valve, with a small distribution at the junction of the left atrial appendage and the anterior wall. The HDF area in case-3 is mainly distributed in the left atrial appendage, posterior wall, and the center of the inferior wall. The HDF area in case-4 is larger, extending in a strip-like fashion from the left superior pulmonary vein ostium and posterior wall all the way to the Septum wall in the direction of the right pulmonary vein, with a scattered distribution in the center of the inferior wall as well. Figure 8B shows the distribution of ablation planning lines in the clinical procedure, and Figure 8C shows the actual ablation target distribution during the surgery. In the data of these four patients, it can be seen that ablation was performed at the recognized HDF sites. For the first three patients, the HDF area in the models were smaller, and the ablation lines passed through the center of the HDF area generated in Figure 8A. For case-4, the HDF area was larger, and the ablation line passed through the strip-like HDF area to cut off the propagation of AF activation.

The distribution of patients regarding the relationship between the left atrial HDF area recognized by the algorithm of this manuscript and the actual surgical ablation is shown in Table 1. Among all 43 patients, there were 3 patients whose 3D DF models showed no significant HDF area, but who had ablation performed outside the pulmonary vein isolation during the surgery. Upon analysis, the Electrophysiologist sought personalized ablation targets based on other AF-related mechanisms, such as low voltage, complex fractionated electrograms (CFAEs), and rotors. There were 7 patients whose models showed HDF area outside the pulmonary vein ostia, but the Electrophysiologist did not ablate them during the surgery. Upon analysis, this was due to the DF values in these patients’ HDF area being lower than the majority of other patients (6.21 ± 0.25 Hz vs. 7.36 ± 0.28 Hz), and ablation was not performed due to a conservative approach to treatment. Overall, HDF areas beyond the pulmonary vein ostia were recognized in 72.1% of PeAF patients, where targeted ablation led to AF termination.

## 4. Discussion

Research indicates that during AF, consistent DF distribution recur within the left atrium, exhibiting a certain degree of organization and regularity [33,34]. This regularity may be associated with changes in the atrial substrate; furthermore, the correspondence between HDF and the origin regions of abnormal atrial electrical activity suggests its potential as an ablation target [35]. This study proposes a method to automatically localize spatiotemporal stability of HDF areas within the heterogeneous and variable electrical activity of the left atrium during AF episodes. The HDF areas extracted by the method in this manuscript are largely consistent with ablation targets beyond the circumferential pulmonary veins during radiofrequency ablation. Notably, AF termination was achieved in the majority of PeAF patients (72.1%) following ablation of these HDF areas, validating the efficacy of the proposed method.

In recent years, several studies have employed artificial intelligence to identify ablation targets for AF. Ref. [36] utilized AI algorithms to map spatiotemporal dispersion (stD) to characterize AF mechanisms from the perspective of electrical activity complexity, finding that stD regions correlate with low-voltage zones and can identify areas of abnormal electrical activity; however, this method essentially focuses on the characterization of electrical dispersion, with limited description of the distribution of atrial electrical activity intensity. Ref. [37] classified unipolar electrograms (EGMs) based on deep learning to identify focal trigger signals; however, the model can only identify whether a segment of EGM is a focal trigger signal and lacks the capability to characterize the overall spatiotemporal distribution of atrial electrical activity. The algorithm proposed in this paper starts from a frequency-domain perspective and focuses primarily on recurring features within left atrial electrical activity. Amidst the heterogeneous and variable electrical activity during AF episodes, it can extract spatiotemporally stable DF distributions to represent the regularity of AF electrical activity. This algorithm provides a novel reliable support for the AF ablation Electrophysiologists, offering reliable basis information for them to locate ablation targets beyond the pulmonary veins, and enriching the information sources for locating ablation targets. From a mechanistic perspective of atrial electrical activity, the spatiotemporally stable HDF areas extracted by the algorithm represent highly active electrical areas within the left atrium. These areas are potentially associated with the sites of origin for abnormal electrical activity or critical re-entry pathways, which are vital for AF ablation therapy. Performing ablation at these sites may facilitate the disruption of the generation and maintenance of abnormal activity, thereby reducing the risk of recurrence resulting from incomplete ablation. Accurate extraction of HDF areas provides Electrophysiologists with auxiliary information on potential ablation areas, facilitating the optimization of ablation strategies.

Regarding the construction of DF distribution models, refs. [24,25] utilized EGM signals collected via sequential mapping to directly calculate DF; however, the resulting models only reflect atrial electrical activity at a single time point. This study proposes a method for calculating spatiotemporal stability of 3D DF distribution models based on EGM signals acquired through sequential mapping during AF ablation. The spatiotemporally stable HDF areas extracted by this method reflect the regular components within abnormal AF electrical activity. Compared to instantaneous features, spatiotemporally stable HDF features provide more robust and reliable auxiliary information for AF ablation. Within the internal architecture of the method, a spatial aggregation algorithm is employed to construct voxel units as the core computational units of the three-dimensional DF model, aggregating discrete electrical activity information collected over long time spans from local tissues into the same voxel unit. This process extracts recurring and stable features to further characterize spatiotemporal stability in DF representations. Within these voxel units, the statistical features of DF value sequences at different discrete time points are calculated to accurately capture recurring atrial electrical activity in the time domain. Furthermore, while ensuring the time-domain stability of DF values, time-domain sparse sampling is implemented to enhance computational efficiency, resulting in an average reduction of approximately 74% in the volume of data processed.

Regarding the calculation of DF values, refs. [19,25] directly applied Fourier transform or Welch spectral estimation to the collected LA-EGM signals to obtain the signal’s frequency domain information and subsequently generated the DF distribution. The accuracy of the results is closely related to signal quality. Ref. [38] calculated the DF value based on Botteron filtering of the LA-EGM signal, which mitigated the impact of noise to a certain extent but still did not resolve issues such as wave fusion and unstable signal periodicity. Building upon the Botteron method, this study introduces an OI threshold to enhance the robustness of the generated DF values against anomalous signals, ensuring that the extracted DF values exhibit sufficient significance. This approach further eliminates interference from harmonics and wave fusion, thereby improving model accuracy.

### 4.1. Analysis of Voxel Size in Spatial Aggregation

Different voxel unit sizes affect the number of coordinate points within the voxel unit. If the voxel unit is too small, the number of coordinate points within it will be too few, reducing the effectiveness of the spatial aggregation method and preventing the extraction of a DF characterization that is stable in the time domain. If the voxel unit is too large, the spatial resolution of the 3D DF model will decrease, affecting the utility of the model. This manuscript analyzes the coordinate point cloud distribution and the constructed 3D DF models under various voxel unit size settings to determine the most suitable size.

In addition to the baseline setting for the voxel unit size used in this manuscript (2 mm), DF models were also calculated with voxel unit sizes set to 1 mm, 3 mm, and 4 mm. The distribution of the coordinate point clouds under different voxel unit size settings is shown in Table 2. Saturated voxel units are defined as those where the number of coordinate points is greater than or equal to 31. Such voxel units can better meet the requirements of spatial aggregation and time-domain sparse sampling by ensuring a sufficient number of points to provide an adequate time span for DF calculation. It can be seen that when the voxel unit size is 1 mm and 2 mm, the difference in the number of effective voxel units is relatively small. However, when the voxel unit size is 1 mm, the total number of voxel units is approximately 7 times that of 2 mm, which would greatly increase the model calculation time. When the voxel unit size is 3 mm and 4 mm, the proportion of effective voxel units compared to 2 mm is not significantly different, but the number of saturated voxel units at 2 mm is much higher than at 3 mm and 4 mm. Saturated voxel units can ensure that the voxel unit contains cardiac electrical activity information over a longer time period, giving the calculated DF better time-domain stability.

**Figure 9:**
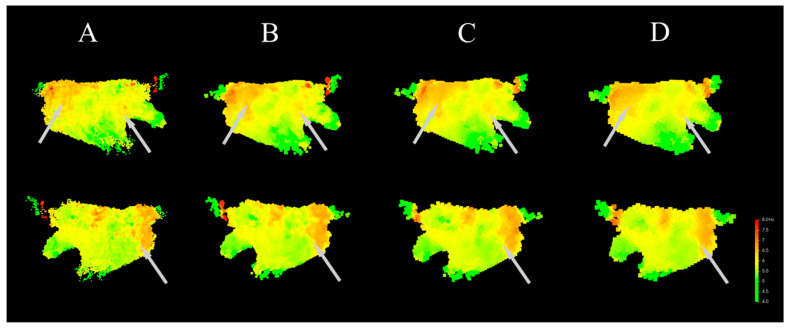
DF models generated with different voxel size (upper side is Anterior-Posterior, lower side is Posterior-Anterior). Gray arrows indicate the regions with significant differences. (**A**) Model generated with voxel size of 1 mm. (**B**) Model generated with voxel size of 2 mm. (**C**) Model generated with voxel size of 3 mm. (**D**) Model generated with voxel size of 4 mm.

The DF models calculated under different voxel unit sizes are shown in Figure 9. It can be seen that the models calculated with voxel unit sizes of 1 mm and 2 mm are similar in terms of DF gradient and the distribution of the HDF area. In the Anterior–Posterior direction, the HDF area is mainly distributed at the junction of the left pulmonary vein and the septum wall, the center of the posterior wall, and sparsely at the junction of the left atrial appendage and the anterior wall. In the models calculated with voxel unit sizes of 2 mm and 3 mm, the model calculated with 2 mm is more refined, and the HDF area at the junction of the left atrial appendage and the anterior wall cannot be recognized in the 3 mm model. As shown in Figure 9, as the voxel unit size increases (i.e., model resolution decreases), the areas with large DF distribution gradients (indicated by the light gray arrows) show gradually reduced gradient distinguishability. That is, as the model resolution decreases, the DF values across large regions tend to become similar, which is detrimental to the model’s ability to distinguish the HDF area. Therefore, given the permissible computational load, a smaller voxel unit size should be selected. In this study, the voxel unit size is set to 2 mm.

### 4.2. Ablation Experiments of Spatial Aggregation

**Figure 10:**
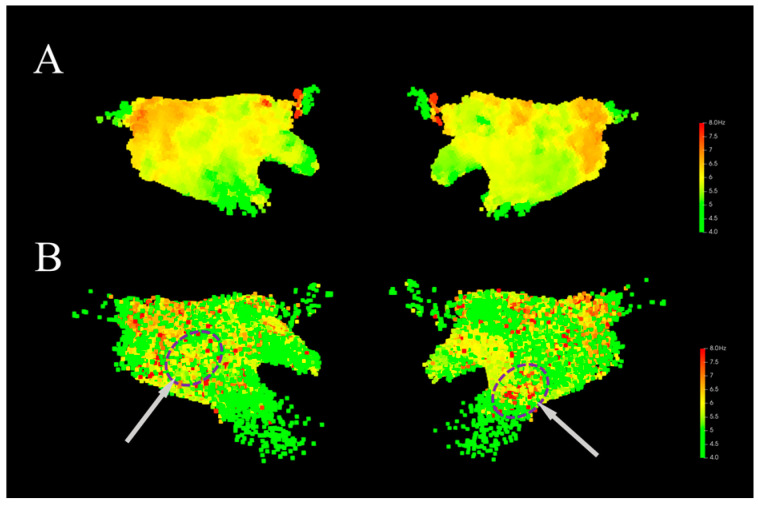
Comparison of 3D DF distribution models generated without using the spatial aggregation method. Gray arrows indicate the locations of abnormal DF sites. (**A**) Model generated with spatial aggregation. (**B**) Model generated without spatial aggregation.

To analyze the effectiveness of the spatial aggregation method, this manuscript generated 3D DF models with settings both utilizing and not utilizing the spatial aggregation method. Figure 10A shows the 3D DF model generated using the spatial aggregation algorithm. It can be seen that in the model, the voxel units are uniformly distributed in space, and the DF values exhibit a distinct gradient distribution, which aligns with the physiological electrical activity of the patient’s atrium during AF. Figure 10B shows the 3D DF model generated directly based on the coordinate point positions. The DF coordinate density distribution is non-uniform, with high coordinate point density and high model resolution in the posterior wall and pulmonary vein ostia, and low model resolution in other locations, particularly near the mitral valve. Furthermore, without the spatial aggregation method, the generated model has a large number of isolated DF outlier points (indicated by the purple circles in Figure 10B), which interfere with the effective identification of the HDF area. These isolated high DF locations only exhibit rapid atrial activation at a single moment. Since this rapid activation does not repeatedly occur, the signal variability is high, and it lacks obvious organization and stability. Some studies have shown that ablation of such HDF area does not yield corresponding therapeutic effects [16,39]. In contrast, the model constructed using the spatial aggregation method incorporates the statistical features of the atrial signal over a long time span at that location, allowing the DF to reflect the potential organization of AF and serve as a potential ablation target. In addition, after applying the spatial aggregation algorithm, it can be combined with the time-domain sparse sampling algorithm, reducing the number of coordinate points for which the DF value needs to be calculated to only about 26% of the original number, which effectively reduces the computational complexity of the model.

### 4.3. Sensitivity Analysis of the OI Threshold

Although Botteron filtering can effectively filter out detailed information in the cardiac electrical activity that is irrelevant to DF extraction, issues such as excessive noise energy or wavefront fusion may lead to low energy for the fundamental wave corresponding to the DF, with harmonic energy being higher than the fundamental. This can result in insufficient significance of the peak detected in the spectrum, leading to the misidentification of a harmonic frequency or another peak as the DF. By setting the OI threshold and filtering out coordinate points whose DF corresponds to an OI value below the threshold, the impact of low DF significance on the 3D DF model can be effectively mitigated. The choice of the OI threshold value significantly influences the experimental results. This manuscript determines a reasonable OI threshold by calculating the corresponding F1 Score under various threshold settings. From all patients’ segment data, 6580 segments were obtained through uniform sampling based on the OI value. Each segment was manually analyzed through its time-domain signal and spectrum to determine if its DF value was sufficiently significant, and a label was assigned to each segment: segments with sufficiently significant DF were labeled as positive samples, and those with insufficient DF significance were labeled as negative samples. The dataset was processed using different OI thresholds, with an interval of $0.05$. Predictions with OI values above the threshold were labeled as positive, and those below were labeled as negative. The F1 score for each OI threshold setting was calculated, as shown in Equations (4)–(6):

(4) $$Pre=\frac{TP}{TP+FP}\;$$

(5) $$recall=\frac{TP}{TP+FN}\;$$

(6) $$F1=\frac{2*Pre*recall}{Pre+recall}$$

where TP (True Positive) denotes positive samples (actual significant DF) with an OI value above the threshold, FP (False Positive) denotes negative samples (actual non-significant DF) with an OI value above the threshold, and FN (False Negative) denotes positive samples (actual significant DF) with an OI value below the threshold.

**Figure 11:**
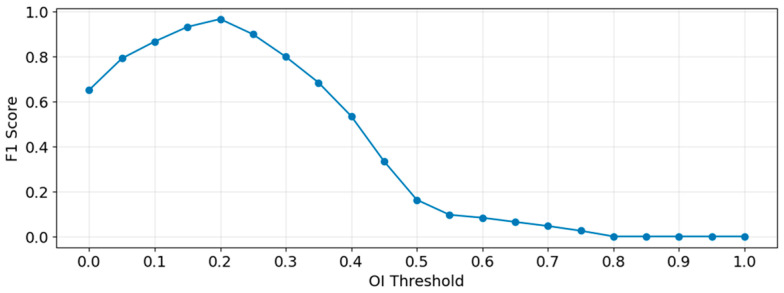
F1 Score under Different OI Thresholds.

The experimental results obtained are shown in Figure 11. It can be seen that the F1-score reaches its maximum value when the OI threshold is 0.2. When the OI threshold is less than 0.2, some DF values whose energy is not sufficiently significant will be introduced into the results, leading to the appearance of outliers in the calculated DF model and affecting the correctness of the model. When the OI threshold is greater than 0.2, as the OI threshold increases, it can largely ensure that the DF values in the results possess sufficient significance, but the number of retained DF values is significantly reduced. This results in a substantial loss of data information in the final model, or even causes some voxel units to become invalid due as they lack a corresponding DF, thereby reducing the spatial coverage of the model. Therefore, 0.2 is chosen as the OI threshold in this experiment.

## 5. Conclusions

Although this study compared the HDF area extracted from PeAF patients with the ablation lines used during the AF ablation procedure, and 72.1% of the ablation targets outside the circumferential pulmonary veins corresponded to the HDF area, which initially validates the effectiveness of the method proposed in this manuscript, the study still has the following limitations: ① Since this study is a retrospective study, the extracted HDF areas were not directly ablated to verify their therapeutic effect on PeAF; moreover, there is a lack of randomness in the distribution of patients’ clinical characteristics, which affects the generalizability of the experimental conclusion. ② The LA-EGM signals in this study were collected using the OCTARAY (OCT) electrode catheter. The authors believe that the method presented in this manuscript would still be effective when using different types of electrode catheters, but the parameter settings at various stages might require adjustment. ③ The number of patients in this study was relatively limited, and there was insufficient information on the clinical characteristics of the patients. The research results require validation with more patient data.

In future research, this study will refer to the experimental design of Chen [40] to conduct prospective trials, aiming to enhance the reliability of the methods proposed in this manuscript. Aligned with the objectives of this study, specialized clinical trials will be designed to investigate the clinical efficacy of spatiotemporally stable HDF areas during ablation procedures in patients with PeAF. Future work will involve expanding the patient dataset to increase clinical diversity and designing randomized controlled trials to further explore the role of spatiotemporally stable HDF areas across different patient populations, thereby improving the generalizability of the experimental findings.

This study proposes a signal analysis and processing scheme for 3D DF models in PeAF patients. By integrating spatial aggregation, LA-EGM signal time-frequency analysis, and local neighborhood spatial filtering, the proposed scheme effectively extracts HDF areas associated with potential ablation targets. Retrospective analysis results demonstrate that in 72.1% of PeAF patients, the extracted HDF areas were consistent with extra-pulmonary vein ablation sites utilized in clinical procedures. The algorithmic model proposed in this manuscript successfully captures recurring electrical activity characteristics of the left atrium during AF; moreover, the resulting spatiotemporally stable HDF areas serve as a reliable basis for the Electrophysiologist to recognize ablation targets beyond the pulmonary veins.

## Figures and Tables

**Table 1:** The distribution of patients regarding the relationship between left atrial HDF area and ablation.

	HDF + Ablation	Without HDF + Ablation	HDF + Without Ablation
Number of Patients	31	3	7
Proportion	72.1%	7.3%	16.3%

**Table 2:** The density distribution of coordinate points with different voxel size.

Voxel Size	Num of Voxel	Num of Full Voxel	Proportion of Effective Voxel
1 mm	148,933	16,100	10.81%
2 mm	24,080	13,819	57.39%
3 mm	8283	5322	64.25%
4 mm	3935	2705	68.74%

## Data Availability

The original contributions presented in the study are included in the article. Further inquiries can be directed at the corresponding author.

## Abbreviations

The following abbreviations are used in this manuscript:

PeAF	Persistent Atrial Fibrillation
LA-EGM	Left Atrial Intracardiac Electrogram
OI	Organization Index
AF	Atrial Fibrillation
PVI	Pulmonary Vein Isolation
HDF	Highest Dominant Frequency
CFAEs	Complex Fractionated Electrograms
